# Genomic biosurveillance of forest invasive alien enemies: A story written in code

**DOI:** 10.1111/eva.12853

**Published:** 2019-09-10

**Authors:** Richard C. Hamelin, Amanda D. Roe

**Affiliations:** ^1^ Department of Forest and Conservation Sciences The University of British Columbia Vancouver BC Canada; ^2^ Institut de Biologie Intégrative et des Systèmes (IBIS) Université Laval Québec QC Canada; ^3^ Département des sciences du bois et de la forêt, Faculté de Foresterie et Géographie Université Laval Québec QC Canada; ^4^ Great Lakes Forestry Center Natural Resources Canada Sault Ste. Marie ON Canada

**Keywords:** forest health, forest management, forestry, genomics, host–parasite interactions, invasive species, molecular evolution, population genetics‐empirical

## Abstract

The world's forests face unprecedented threats from invasive insects and pathogens that can cause large irreversible damage to the ecosystems. This threatens the world's capacity to provide long‐term fiber supply and ecosystem services that range from carbon storage, nutrient cycling, and water and air purification, to soil preservation and maintenance of wildlife habitat. Reducing the threat of forest invasive alien species requires vigilant biosurveillance, the process of gathering, integrating, interpreting, and communicating essential information about pest and pathogen threats to achieve early detection and warning and to enable better decision‐making. This process is challenging due to the diversity of invasive pests and pathogens that need to be identified, the diverse pathways of introduction, and the difficulty in assessing the risk of establishment. Genomics can provide powerful new solutions to biosurveillance. The process of invasion is a story written in four chapters: transport, introduction, establishment, and spread. The series of processes that lead to a successful invasion can leave behind a DNA signature that tells the story of an invasion. This signature can help us understand the dynamic, multistep process of invasion and inform management of current and future introductions. This review describes current and future application of genomic tools and pipelines that will provide accurate identification of pests and pathogens, assign outbreak or survey samples to putative sources to identify pathways of spread, and assess risk based on traits that impact the outbreak outcome.

## 1 INTRODUCTION

Natural, planted, and urban forests play a vital role in the world economy and provide important cultural and socioeconomic benefits. Healthy forests support a healthy environment by providing ecosystem services that range from carbon storage, nutrient cycling, and water and air purification, to soil preservation and maintenance of wildlife habitats (Tallis, Kareiva, Marvier, & Chang, 2008). Increasingly, studies show that human health outcomes are correlated with the area covered by forests (Maller, Townsend, Pryor, Brown, & St Leger, 2006).

Climate change and invasive alien species threaten valuable natural and planted forest resources and can be responsible for large and irreversible damage to forest ecosystems that would jeopardize their capacity to provide long‐term fiber supply and ecosystem services (Loo, 2009; Simberloff et al., 2013; Trumbore, Brando, & Hartmann, 2015; Wingfield, Brockerhoff, Wingfield, & Slippers, 2015). The global spread of invasive species has intensified in the last century, resulting in unprecedented alterations in the distribution of the earth's biota (Fisher et al., 2012; Mack et al., 2000). This trend is directly related to anthropogenic activities, brought about largely by the dramatic increase in global trade and the movement of people and goods (Hulme, 2009; Westphal, Browne, MacKinnon, & Noble, 2008). In fact, there is a strong influence of economic and demographic factors on the levels of invasion by alien species (Hulme, 2009; Pysek et al., 2010; Santini et al., 2012). This increase in the global movement of biota, combined with a changing climate, creates new opportunities for invasive species to expand their range, establish in new environments, and hybridize and develop novel adaptations (Colautti & Lau, 2015; Depotter, Seidl, Wood, & Thomma, 2016; Fisher et al., 2012; Stenlid & Oliva, 2016).

The key to reducing the threat from forest invasive alien species is via vigilant biosurveillance. This process of gathering, integrating, interpreting, and communicating essential information about pest and pathogen threats is aimed at prevention, early detection, and improved decision‐making (Anonymous, 2012; Epanchin‐Niell & Liebhold, 2015; Poland & Rassati, 2019; Roe et al., 2018). We urgently need efficient systems for global biosurveillance of invasive species to increase preparedness and facilitate early interventions, stopping invasions at the early stages of arrival. This early intervention translates into increased likelihood of stopping a potential invasion and is cost‐effective (Bilodeau et al., 2018; Liebhold & Tobin, 2008; Lovett et al., 2016; Yemshanov et al., 2017). For example, in Toronto, Canada, early detection of Asian long‐horned beetle led to a rapid and aggressive response to the invasion, resulting in eradication of the invasive populations (Fournier & Turgeon, 2017; Smith, Turgeon, Groot, & Gasman, 2009). Conversely, the cost of managing or eradicating forest invasive species increases dramatically over time as containment fails and management efficiency decreases (Pimentel, Lach, Zuniga, & Morrison, 2000). The emerald ash borer, for example, was not detected during early stages of invasion and has now spread throughout eastern North America. It is considered one of the most costly and destructive invasive forest pests in North America and may lead to the loss of an entire genus of trees (Herms & McCullough, 2014; Kovacs et al., 2011; McKenney et al., 2012).

Conducting biosurveillance is challenging (Bilodeau et al., 2018). The diversity of pests and pathogens that threaten forests requires a breadth of taxonomic expertise that is very difficult to acquire and maintain. In addition, pests and pathogens are disseminated at different life stages, complicating their rapid and accurate identification. Invasive alien species can enter via a variety of pathways, creating uncertainty about their origin and likely pathways of introduction, thereby complicating decision‐making regarding resource allocation, mitigation, and management. An additional difficulty is that alien species have varying abilities to become established in a novel environment, making risk assessment an exercise that relies upon educated guesses.

Genomics can provide powerful new solutions to these challenges (Roe et al., 2018). The process of invasion is a story written in four chapters: transport, introduction, establishment, and spread. The series of processes that lead to a successful invasion leave behind a DNA signature that tells the story of the invasion. This signature can help us understand the dynamic, multistep process of invasion (Blackburn et al., 2014; Gladieux et al., 2015; Garnas et al., 2016; Renault, Laparie, McCauley, & Bonte, 2018) and inform management of current and future introductions.

Reading this story requires generating genomic resources. Most invasive species are nonmodel organisms that lack pre‐existing genomic resources. Therefore, new targeted approaches are needed to rapidly develop foundational genomic knowledge and tools for nonmodel invasive species. For the purposes of this review, we focus on exotic or alien species, although many of these tools and processes apply to native insects that could become invasive due to anthropogenic global change. Our review will describe current and future applications of genomic tools and analyses that can provide accurate identification of pests and pathogens, assign intercepted samples to putative sources to identify pathways of spread, and assess risk based on traits that impact the outbreak outcome (Bilodeau et al., 2018). We will provide examples of some of the most important alien invasive species of trees and forests, and we will also highlight some key examples of the use of genomic biosurveillance on native pests that have recently become invasive. We will also identify the key technical challenges as well as the hurdles in the implementation of these tools. Finally, we will discuss some of the solutions to these challenges and examine the exciting future outlook of genomic biosurveillance of forest invasive species.

## 2 INVASION BY ANY OTHER NAME

The rapid and accurate identification of biological samples collected during surveys or inspections is an essential first step in biosurveillance. Typically, surveys are targeted activities designed to sample a specific area to assess the presence of one or several potential invasive species. For example, pheromone‐baited traps are used to monitor for the presence of the Asian gypsy moth, a potential invasive species around ports of entry in North America and Europe (Liebhold, MacDonald, Bergdahl, & Mastro, 1995). For pathogens, baiting methods are commonly used to assess the species composition of oomycetes and monitor the presence of the sudden oak death pathogen in natural forests (Sutton, Hansen, Reeser, & Kanaskie, 2009) or in high‐risk watersheds downstream from infected nurseries (Oak, Hwang, Jeffers, & Tkacz, 2010). Surveys are conducted for diseases caused by fungal pathogens, such as the oak wilt, which is present in the US states bordering Canada, but not found in Canada, by looking for typical symptoms and signs, including the typical fungal mats. By contrast, the routine inspections at ports of entry aim to systematically search for any suspicious biological material in imported goods that could represent a risk. Given the taxonomic breadth that can be encountered during these activities, it is almost impossible to perform reliable on‐site accurate identification. This task is complicated by the different life stages encountered during surveys and inspections: eggs, larvae or adults for insects and spores, and fruiting bodies or mycelium for fungi and oomycetes. Even when samples are taken to the laboratory, accurate identification can be difficult. The presence of subspecies or sister species and the paucity of morphological characters compound this problem. The flux in fungal and insect taxonomy and the seemingly endless task to discover, describe, and name the vast diversity in the world are additional challenges (Godfray, 2002; Hibbett & Taylor, 2013).

### 2.1 One locus to rule them all

The simple and elegant idea that a short universal DNA fragment can be used to classify living organisms is a revolutionary concept that finds extremely useful applications in the context of documenting biodiversity (Hebert, Cywinska, Ball, & deWaard, 2003). Application and use of DNA barcoding have proved effective in forest pest identification and biosurveillance and are well described (Bilodeau et al., 2018). The genetic variation comprised within the DNA barcodes has also been widely translated into taxon‐specific rapid and sensitive detection assays using polymerase chain reaction (PCR) (reviewed in Martin, James, & Levesque, 2000; Vincelli & Tisserat, 2008) and applied to invasive species surveillance and management (Armstrong & Ball, 2005). There are several well‐established operational applications of such assays targeting some of the most threatening forest diseases and insects (Lamarche et al., 2015; Martin et al., 2009; Stewart et al., 2016) that demonstrate the potential of DNA‐based detection of invasive species. Application of DNA detection of invasive insects such as the Asian gypsy moth in bulk samples collected in pheromone traps promises to generate cheap and very efficient monitoring approaches (Stewart et al., 2019).

Despite the practicality of a single DNA barcode, this approach has drawbacks (Dupuis, Roe, & Sperling, 2012; Mallo & Posada, 2016; Moritz & Cicero, 2004; Taylor & Harris, 2012) and can fail to identify new species (Hickerson, Meyer, & Moritz, 2006; Vyskocilova, Tay, Brunschot, Seal, & Colvin, 2018) or those with complex evolutionary histories (Percy et al., 2014). In fungi, DNA‐barcoding genes can vary in their diagnostic efficiency among taxonomic groups (Gao & Zhang, 2013; Roe, Rice, Bromilow, Cooke, & Sperling, 2010; Vialle et al., 2009). Thus, a more nuanced diagnostic framework is needed. Additional loci consistently increase delimitation success across a wide range of taxa, supporting a multilocus integrative approach (Dupuis et al., 2012). Multilocus DNA barcoding that combines cytochrome oxidase 1 and ribosomal RNA from the internal transcribed spacer (ITS) or the large subunit (28S) has proven successful in identifying fungal and oomycete plant pathogens (Feau, Vialle, Allaire, Maier, & Hamelin, 2011; Robideau et al., 2011) and discovering misidentified plant pathogens in public collections, which are critical references for identifying new potential invasive species (Feau et al., 2009).

**Figure 1:**
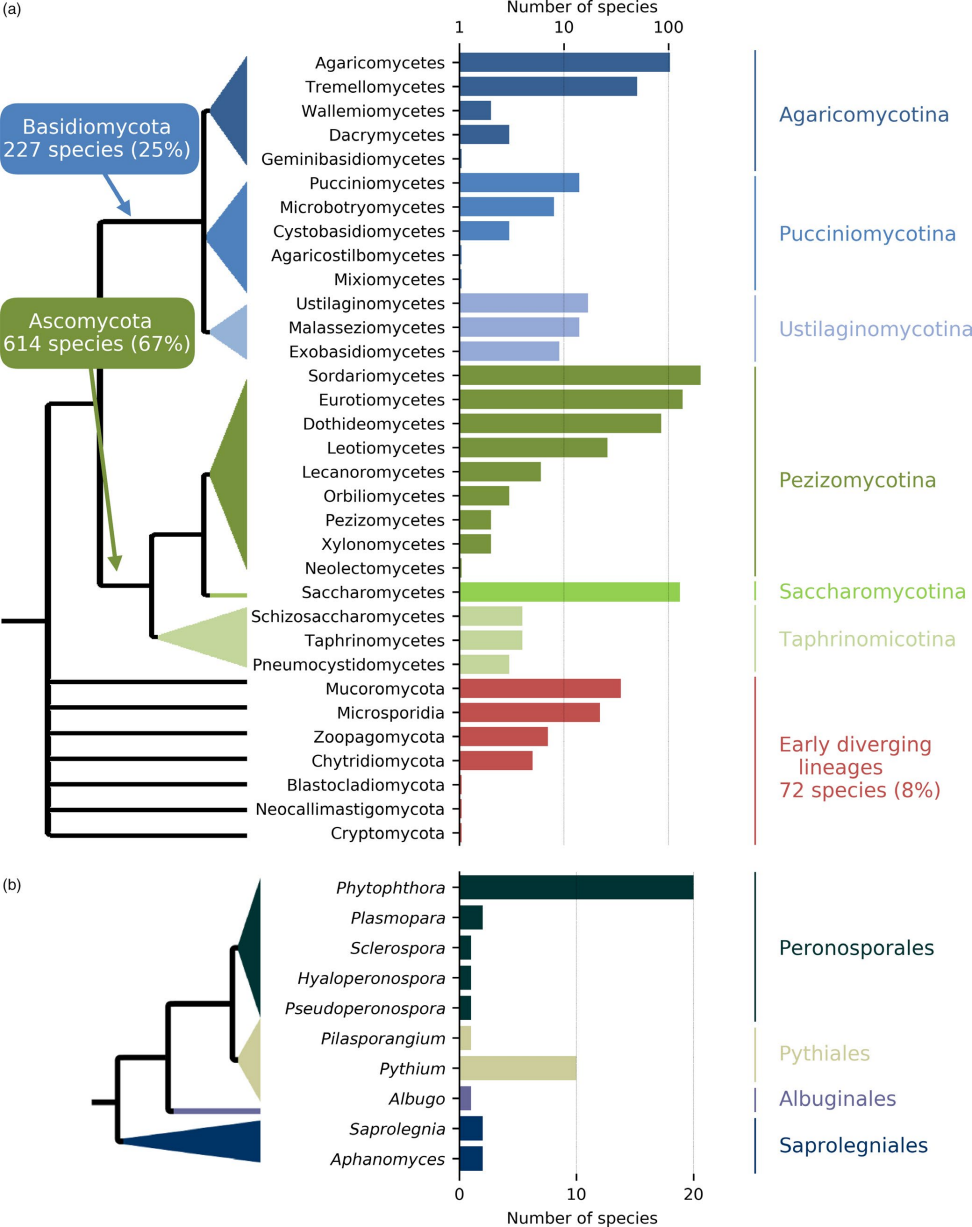
Number of species and phylogenic coverage of fungal (a) and oomycete (b) genomes available on the NCBI public database (accessed on November 2017). Reproduced courtesy of Nicolas Feau and PeerJ (Feau et al., 2018)

Whole‐genome sequencing and phylogenomic approaches make it possible to go beyond the DNA‐barcoding genes and search entire genomes to resolve evolutionary patterns and identify discriminant regions that can become diagnostic. Phylogenomics has been applied to insects and fungi by analyzing single‐copy genes extracted from genome sequences and classified into clusters of orthologs (Marthey et al., 2008; Misof et al., 2014). The 1,000 fungal genome initiative is generating whole genomes across multiple fungal lineages that have been used to generate a genome tree of life for the fungal kingdom (Choi & Kim, 2017; Grigoriev et al., 2013). These genomes are useful to improve our ability to recognize and name fungi. A genome‐enhanced detection and identification pipeline used genomes of fungi and oomycetes (Figure 1) to identify discriminant genome regions (Bergeron, Feau, Stewart, Tanguay, & Hamelin, 2019; Feau et al., 2018; Lamarche et al., 2015). The discriminant genome regions can be easily multiplexed to generate multilayered assays that provide both redundancy and increased taxonomic resolution (Feau et al., 2018). This pipeline can be applied to a diverse range of forest invasive species as whole‐genome sequences become accessible in more organisms.

### 2.2 What's in a name?

In the management and control of invasive species, defining and identifying species are critical given that this is the evolutionary unit tied to regulatory policies and management. Defining and delimiting species is challenging. Even trying to answer the question “what is a species?” is fraught with controversy and disagreement (Zachos, 2016). Entomologists continue to grapple with this question (Claridge, 2017). In fungi, there is an additional challenge caused by the dual system of nomenclature still frequently used for the sexual and asexual phases of pleomorphic fungus species (Hibbett & Taylor, 2013). A “one fungus‐one name” approach was proposed as a solution to resolve the dual taxonomy system (Taylor, 2011; Wingfield et al., 2012) and was included in the rules of International Code of Botanical Nomenclature (McNeill & Turland, 2011). Sequence‐based classification and identification in fungi and changes to nomenclatural rules to allow sequence‐based taxon descriptions should contribute to widespread applications of this principle (Hibbett & Taylor, 2013).

A phylogenetic approach that recognizes fungal species based on the concordance of multiple gene genealogies was proposed (Taylor et al., 2000). This method has been applied to fungi to define species boundaries in the absence of clear morphological traits (Alamouti et al., 2011; Cai et al., 2011; Sakalidis, Hardy, & Burgess, 2011; Vialle, Feau, Frey, Bernier, & Hamelin, 2013). In a biosurveillance context, this matters as some of those previously unidentified taxa have different host ranges; therefore, accurate taxonomic identification can reveal the likely host, which in turn can impact outbreak predictions. Although the practical implications of accurate taxonomic classification and identification are clear, the next challenge will be to define sequence‐based classification standards so that the plant health community and the regulatory bodies can agree on names (Hibbett, Ohman, & Kirk, 2009; Hibbett & Taylor, 2013). It may also be that the evolutionary unit of invasion is not a species, but rather a population. For example, if a population of a native species becomes invasive in response to anthropogenic change (Carey, Sanderson, Barnas, & Olden, 2012) then, delimitation of the invasive unit will (if possible) require sequence‐based classification. The advantage of genomic approaches is the ability to clearly define populations, and may permit the identification of potentially cryptic invasives relative to native populations, particularly if spread occurs into novel habitats.

### 2.3 The universe in a grain of sand

DNA barcoding is transitioning from barcoding individuals to barcoding entire communities, known as metabarcoding (Cristescu, 2014). Metabarcoding can generate large numbers of DNA barcodes directly from environmental samples by amplifying DNA using primers in conserved regions, usually within the DNA‐barcoding genome regions and performing high‐throughput sequencing (HTS) of the amplified product. It is particularly powerful to determine taxa composition in communities, establish baseline data, and discover unknown or undescribed taxa. This can be used for the identification of non‐native species at several steps along the introduction process, informing the control and management of potential non‐native species (Comtet, Sandionigi, Viard, & Casiraghi, 2015). Metabarcoding has been applied to bulk samples of arthropods to reconstruct community membership and compare biodiversity (Yu et al., 2012) and to detect invasive species in the Great Lakes from environmental DNA (Klymus, Marshall, & Stepien, 2017).

Metabarcoding has strengthened our ability to document biodiversity and had a particularly profound impact on our knowledge of the less visible components of our ecosystems, such as fungi and oomycetes (Hibbett et al., 2009). The unexpectedly large fungal diversity found in soil samples and the large proportion of unidentified taxa point to the challenges and opportunities that lie ahead in the study of fungal biodiversity (Buée et al., 2009). In the context of invasive species, metabarcoding can be a powerful approach to generate both baseline data and early warning of plant diseases and pests (Abdelfattah, Malacrinò, Wisniewski, Cacciola, & Schena, 2018; Bérubé & Nicolas, 2015). Metabarcoding surveys of soil and roots can reveal the presence of broad collections of pathogenic species in multiple phylogenetic clades and often include multiple unique and new records (Bose, Wingfield, Roux, Vivas, & Burgess, 2018; Burgess et al., 2017; Català, Berbegal, Pérez‐Sierra, & Abad‐Campos, 2017; Prigigallo et al., 2016). Metabarcoding can become an important tool in the invasion toolkit (Comtet et al., 2015; Kamenova et al., 2017) and has been used in surveillance of insects and associated plant and human pathogens (Batovska et al., 2018). It has helped uncover novel vector–pathogen relationships (Malacrinò et al., 2017), as well as new parasitoid assemblages in invasive species (Kitson et al., 2019). Trees can be viewed as ecosystems in themselves harboring a diversity of organisms (Feau & Hamelin, 2017). A signature of tree health can be obtained by recording shifts in the microbiome composition of a tree in response to infection by invasive pests and pathogens (Koskella, Meaden, Crowther, Leimu, & Metcalf, 2017). Deciphering the pathobiome, the pathogenic agents integrated within its biotic environment, can reveal interaction networks that can impact pathogen establishment (Jakuschkin et al., 2016; Vayssier‐Taussat et al., 2014). Metabarcoding provides greater resolution to the biological complexity that underlies these novel systems than traditional approaches, potentially giving us a better understanding of the process of invasion.

To fully realize the power of metabarcoding approaches, it is necessary to address several challenges. First, metabarcoding is dependent on validated reference libraries. As with any molecular diagnostic approach, DNA sequences from samples obtained during surveys or inspections are compared to a collection or library of known, previously identified samples. The goal is to identify the best or closest match to a DNA sequence of an unknown sample. The two possible outcomes of searching a database with a query sequence are (a) a perfect match, which provides a likely taxonomic identification, and (b) a mismatch which could reveal a new species or subspecies or at least provide some information on the taxonomic placement of the unknown. Substantial work is required to develop validated reference libraries. Validation requires the assessment and verification of all known samples by taxonomic experts to ensure that the name attached to the reference sample is accurate (Geiger et al., 2016). This process is time‐consuming, and yet critical to the success of these methods.

This process can be challenging for fungi and oomycetes where the paucity of data on voucher specimen restricts the usefulness of the references. To help address this problem, the UNITE database provides high‐quality records for ITS sequences for vouchered specimens deposited in herbaria and identified by contributors (Kõljalg et al., 2005). Recently, a concerted effort identified high‐quality reference sequences and re‐annotated poorly annotated public ITS sequences in plant pathogenic fungi (Nilsson et al., 2014). Another database that links scientific names, reference specimens, and molecular data for fungi (the Fungal Internal Transcribed Spacer RNA [ITS] RefSeq Targeted Loci Project) uses the well‐established NCBI platform and focuses on sequences from the ITS derived from type specimens and/or ex‐type cultures (Schoch et al., 2014). But even with the current 70,000 taxa in the database, it represents <5% of the estimated global fungal diversity (Hawksworth, 1991).

Insects lack comparable databases, although the International Nucleotide Sequence Database Collaboration (INSCD = GenBank, EMBL, DDBJ) and Barcode of Life Database embody the centralized genomic reference libraries for this group. Collectively, these databases contain an astonishing volume of data, although the consistency of validated records is highly variable. In fact, many new entries lack taxonomic names (let alone names validated against a voucher specimens), so‐called “dark taxa” (Page, 2016). Species description and validation of dark taxa continue to lag behind the rapid generation of new sequence data (Page, 2016). For fungi, a “nomenclatorial crisis” is caused by the large environmental sequencing efforts, which generate DNA‐barcoding sequence data without a physical specimen (Taylor, 2011). In the context of invasive species, these unknown and unnamed samples represent a challenge: Is an unidentified sample a new potential invasive, an undescribed endemic taxon, or an unsampled native species?

Additional methodological biases can yield artificial results and misleading conclusions (Lindahl et al., 2013; Scott et al., 2018). In particular, some of the early claims of astonishingly high species richness in 454‐sequenced amplicons were likely exaggerated because of problems in distinguishing technical artifacts from true diversity. Also, biases in the “universal” primers used in metabarcoding fungi have been highlighted *in silico* (Bellemain et al., 2010). Certainly, the development of methods and databases has helped the fungal research community apply metabarcoding to various research questions (Kõljalg et al., 2005; Lindahl et al., 2013). But the important question is whether the presence of a short fragment of DNA of an invasive species in a metabarcoding survey is sufficient to confirm the presence of an invasive species and put in place management and mitigation measures. Currently, the use of metabarcoding within a regulatory framework remains marginal until these challenges are adequately addressed.

## 3 A STORY WRITTEN IN CODE

Determining the geographic origin and route of invasion is important in resolving an invasive species’ history, (Cristescu, 2015). Also known as invasion forensics, this process can help identify high‐risk regions and guide management or trade practices to limit the movement of future invasive species (Chown et al., 2015). Identifying the center of diversity of an invasive species also provides critical guidance for the development of management methods, for example in the collection and development of biological control agents (Hoelmer & Kirk, 2005), to identify genetic resistance in the host (Smalley & Guries, 1993) and to prevent additional introductions or re‐introductions of more aggressive subspecies (Brasier, 2000a).

Genomic data provide a path to resolving the complex story of invasion (Cristescu, 2015; Estoup & Guillemaud, 2010; Rius, Bourne, Hornsby, & Chapman, 2015). We are now, more than ever, able to read this story written in code. Genomic data can be used to identify invasion sources and routes of introduction by assigning intercepted samples to populations in the native range. The number of introduction events, patterns of colonization and establishment, range expansion, bottlenecks, bridgeheads, lag times, and adaptation to novel environments all leave population genomic signatures that can be extracted to document and understand the invasion process (Chown et al., 2015; Cristescu, 2015; Sakai et al., 2001). The capacity to rapidly generate vast quantities of genomic data or even entire genomes is changing how we can approach invasion genetics and genomics.

### 3.1 The roads most travelled: identifying origins and pathways

Genomics provides a powerful, albeit indirect, approach to reconstructing probable invasion scenarios (Estoup & Guillemaud, 2010). By first characterizing the genomic variation in an organism's native range and geographically mapping the diversity, it is possible to assign invasive populations or intercepted specimens to known source populations (Chown et al., 2015; Manel, Gaggiotti, & Waples, 2005) or characterize routes of invasion (Estoup & Guillemaud, 2010). This approach has been used to identify the source or pattern of introduction of invasive exotic forest insects (Lesieur et al., 2018; Picq et al., 2018; Taerum, Konecny, Beer, Cibrian‐Tovar, & Wingfield, 2016) and pathogens (Barres et al., 2012; Bergeron et al., 2011; Brar et al., 2015; Dutech et al., 2012; Goss, 2015; Hamelin et al., 2000; Hamelin, Lecours, & Laflamme, 1998; McMullan et al., 2018; Sakalidis, Feau, Dhillon, & Hamelin, 2016) or even native pests that become invasive due to anthropogenic changes (Janes et al., 2014; Trevoy, Janes, & Sperling, 2018).

There are multiple methods to analyze genome‐wide data to delineate groups or clusters and identify individuals that share common ancestors and geographic origins (reviewed in Alhusain & Hafez, 2018; Lawson & Falush, 2012; Manel et al., 2005). In an analytical workflow, we would first examine the population structure using exploratory methods without *a priori* assumptions about genetic structuring such as principal component analyses or discriminant analyses to assess patterns in the population data, followed by clustering and assignment. One of the challenges is that the size of datasets generated by global population genomics will require increasing computing capacity and result in longer processing times to perform assignment analyses. Promising new avenues are emerging that streamline classification or assignments of genomic data (Beugin, Gayet, Pontier, Devillard, & Jombart, 2018; Jombart, Devillard, & Balloux, 2010; Jombart, Eggo, Dodd, & Balloux, 2011). As genomic biosurveillance strives toward real‐time, field‐based screening (see Future Directions), the field will need computationally efficient analytical approaches that can deliver identifications in short time frames.

Accurate demarcation of population structure is dependent upon adequate sampling throughout an invasive species range. Sampling must be conducted at the appropriate scale and will be influenced by the underlying population genomic variability and structure. Population assignment is more accurate if native populations are highly structured compared to those that are panmictic. Extensive and contemporary migration among geographic areas within the native range can further blur the boundaries between populations and reduce the likelihood of accurate source assignment (Carter, Smith, & Harrison, 2010; Lombaert et al., 2011). This problem is compounded in species with asexual reproductive cycles. In some tree pathogens, the sexual cycle is absent or rare and global populations are comprised of just a few clonal lineages such as the sudden oak death pathogen *Phytophthora ramorum* (Grünwald, Everhart, Knaus, & Kamvar, 2017; Grünwald, Garbelotto, Goss, Heungens, & Prospero, 2012; Grünwald et al., 2009). Yet, populations rarely reach complete genetic homogeneity, so the use of genomic approaches should help resolve such issues (Cristescu, 2015). For example, whole‐genome population re‐sequencing is revealing a much greater genomic diversity in asexual lineages of *P. ramorum* than previously observed, with aneuploidy and mitotic recombination generating new multilocus genotypes that could be useful for population delimitation and assignment (Dale et al., 2019; Elliott et al., 2018; Kasuga et al., 2016).

Given that much of the biological complexity of invasive systems is not known *a priori*, it is necessary to build global reference collections that capture as much variability as possible. Exhaustive sampling is impossible, so one must decide on an effective sampling design given limited knowledge. Minimum sample sizes have been proposed, typically between 25 and 30 individuals per population (Hale, Burg, & Steeves, 2012), but can be as low as eight in some cases (Nazareno, Bemmels, Dick, & Lohmann, 2017). Recent work on genetic conservation presents a simulation method that could direct sampling designs using a data‐informed model based on kinship and pedigree analysis (Flesch, Rotella, Thomson, Graves, & Garrott, 2018). But we still lack a similar methodology that focuses on population structure determination and downstream assignment.

A bigger problem is that we do not even know the native range of some of the most invasive and damaging forest pathogens (Brasier & Mehrotra, 1995; Grünwald et al., 2012), let alone their native population structure. Pathogens causing Dutch elm disease and sudden oak death have broad distributions on more than one continent and may occupy different niches in their center of origin or exist as cryptic species that do not cause the same visible diseases as in their invasive ranges. In such cases, sampling the native range is simply not possible. But even in the absence of knowledge of the native range of an invasive species, genomic analyses of populations can reveal the postestablishment dissemination pathways in the invasive range or estimate the original number of introduced individuals (Grünwald et al., 2012; Grünwald, LeBoldus, & Hamelin, 2019). This information could still be useful to prevent secondary spread and identify pathways of dissemination.

The application of whole‐genome sequencing in invasive species biology is changing our approaches to population characterization. Earlier work on invasive species used a combination of mtDNA and/or microsatellites to characterize source populations in insects and pathogens (Boissin et al., 2012; Carter, Smith, Turgeon, & Harrison, 2009; Dutech et al., 2012; Garbelotto, Guglielmo, Mascheretti, Croucher, & Gonthier, 2013; Gross, Hosoya, & Queloz, 2014; Havill et al., 2016; Schoebel, Stewart, Gruenwald, Rigling, & Prospero, 2014). This combination of markers has shown some success resolving invasion history (Cristescu, 2015), but these coarse markers often lack the resolution to clearly resolve invasion routes, particularly in complex scenarios (Carter et al., 2010; Javal et al., 2017, 2019) and cannot capture the fine‐scale demographic or adaptive processes that accompany complex invasion dynamics (Cristescu, 2015). The developments of HTS and genome‐wide sampling strategies yield rich single nucleotide polymorphism (SNP) datasets that provide a detailed snapshot of an organism's genome‐wide diversity and can provide high‐resolution documentation of the invasion process (Cristescu, 2015).

Genome sequencing advancements in the past decade have greatly expanded access to genomic data for nonmodel organisms (Goodwin, McPherson, & McCombie, 2016). Whole‐genome sequences and resources are now available for a wide number of forest invasive insects (reviewed in Roe et al., 2018) and fungi (Feau et al., 2018; Grigoriev et al., 2013), including forest pathogens (Dhillon et al., 2015; Duplessis et al., 2011; Feau et al., 2016; Ohm et al., 2012; Olson et al., 2012; Tyler, 2006). For fungi, the small size of the genomes (averaging 37, 46, and 75 MB in ascomycetes, basidiomycetes, and oomycetes, respectively) makes HTS of multiple samples a viable option and provides a powerful way to obtain a complete picture of an organism's genomic variation and architecture (Grünwald, McDonald, & Milgroom, 2016). Insects have larger genomes (up to 1 GB), so HTS at the population level is still unfeasible and genome sampling strategies such as reduced representation libraries (Altshuler et al., 2000; Baird et al., 2008; Elshire et al., 2011) are often used.

Genome‐wide approaches provide dense marker sampling and much greater resolution than traditional markers, such as mitochondrial DNA and microsatellites (Rasic, Filipovic, Weeks, & Hoffmann, 2014; Santure et al., 2010; Vali, Einarsson, Waits, & Ellegren, 2008). Genome‐wide SNPs are more powerful than microsatellites at assigning unknown samples to source populations (Puckett & Eggert, 2016) and can resolve subtle genetic differences between populations that can be incorrectly described as admixture with microsatellites (Dupuis et al., 2017). This can impact the inference of population structure in a genomic reference map (Fischer et al., 2017; Haasl & Payseur, 2011; Liu, Chen, Wang, Oh, & Zhao, 2005; Munoz et al., 2017) and subsequent assignment of unknown samples to a source population (Puckett & Eggert, 2016). In addition, SNPs are more amenable to downstream applications as they can be mapped onto a reference genome and can serve as a foundation for trait characterization (see below) and diagnostic assay design, critical for the development of point‐of‐need assays.

### 3.2 Population assignment and beyond

Beyond characterizing invasion sources and routes, genomic profiles can reveal additional characteristics of the invasion process during the establishment and spread phases. Population genomics is revolutionizing the identification of adaptive molecular variation and the estimation of important parameters such as population size and migration rates (Luikart, England, Tallmon, Jordan, & Taberlet, 2003). Whole‐genome sequencing can be used to assess the size of the founding and source populations and the adaptive potential in the invasive population. Whole‐genome sequencing of *Hymenoscyphus fraxineus*, the causal agent of ash dieback, revealed that the European population of this invasive pathogen was founded by two divergent haploid individuals from a large source population. The adaptive diversity in genes involved in host–pathogen interaction, such as effectors, was maintained in the introduced population but was far greater in the native range, prompting the need to prevent additional introductions (McMullan et al., 2018).

Multiple introductions via complex global movement could be more common in the invaded range than previously thought (Garnas et al., 2016). Divergent lineages within invasive populations can indicate multiple independent introductions (Hamelin et al., 1998) and are often detected in successful invasions (Dlugosch, Anderson, Braasch, Cang, & Gillette, 2015; Dlugosch & Parker, 2008). Multiple introductions may be indicative of high levels of propagule pressure (Lockwood, Cassey, & Blackburn, 2009; Simberloff, 2009), which, minimally, represents repeated breaches of the regulatory system. Invasive populations derived from multiple sources or via multiple introduction events can have higher genetic diversity, which is thought to promote invasiveness (McDonald & Linde, 2002). However, there is a genetic paradox of invasions and many successful invaders do not follow a simple pattern of diversity. Founder events could actually play a beneficial role by purging harmful alleles or by increasing additive genetic variance (Garnas et al., 2016). It has also been argued that in fungi the huge census size allows adaptation even in bottlenecked, clonal invaders (Gladieux et al., 2015).

Multiple introductions can also facilitate further evolutionary change via admixture, hybridization, or horizontal gene transfer between divergent lineages (Ellstrand & Schierenbeck, 2000; Hufbauer, Rutschmann, Serrate, Vermeil de Conchard, & Facon, 2013; Keller & Taylor, 2010), which can alter their evolutionary trajectories (Keller & Taylor, 2010; Rius & Darling, 2014; but see Barker et al., 2019). These processes can lead to adaptive evolution (Feurtey & Stukenbrock, 2018) via the acquisition of new traits that could affect the success or failure of an invasive species (Brasier, 2000b) and, by extension, alter the invasion risk posed by a particular population. For example, the aggressive pathogen that causes the Dutch elm disease (*Ophiostoma novo‐ulmi*) initially reproduced only clonally via asexual propagation, but then gained the ability to reproduce sexually by acquiring a mating‐type gene from the less aggressive *O* *. ulmi* (Paoletti, Buck, & Brasier, 2006). The ability to recombine sexually is viewed as an important trait in invasive plant pathogens (McDonald & Linde, 2002; Philibert et al., 2011). It could be a critical factor in the invasive success of *O. novo‐ulmi* that has led to the near extinction of elms in eastern North America. Horizontal gene transfer could also increase pathogenic potential in invasive species. The poplar pathogen *Sphaerulina musiva* acquired, via horizontal transfer, genes that are important to the pathogen's ability to attack woody tissues; this could have conferred to this fungus the ability to cause stem cankers, a type of injury that greatly reduces the tree's growth potential and is often lethal (Dhillon et al., 2015).

### 3.3 Risky business: predicting invasiveness with genomics

Risk assessment is an important part of evaluating the threat of pests and pathogens and prioritize prevention and mitigation resources (Bilodeau et al., 2018). Knowledge and prediction of traits associated with successful invasions (e.g., virulence, host range, or thermal tolerance) using genomics would be highly informative and could help predict the risk of establishment and spread of potential invasive species (Chown et al., 2015). Adaptation genomics aims at determining the genetic architecture of a trait important in terms of adaptation. In fact, it consists of linking phenotype with genotype (Stapley et al., 2010). An adaptive trait (and associated loci) in an invasive species is one that has enabled or facilitated the establishment and spread into novel habitats, and confers a fitness advantage in the novel environment (Colautti & Lau, 2015). Identifying genomic regions that are associated with “invasiveness” is considered one of the greatest challenges in invasion genetics (Losos et al., 2013; Roe et al., 2018). Establishing this causative link is not trivial. Major challenges still exist in linking phenotypic changes in invasive populations with simultaneous changes in the genome (Hoban et al., 2016; Sork, 2017; Stapley et al., 2010).

New powerful approaches driven by whole‐genome sequencing have been developed to identify genes under selection and those associated with adaptive traits (Vitti, Grossman, & Sabeti, 2013). Genome‐wide association studies (GWAS) can be used to find markers associated with adaptive traits (Santure & Garant, 2018). This approach was used to discover SNPs associated with virulence and to identify known and novel virulence genes in the conifer pathogen *H. annosum* (Dalman et al., 2013). There are additional examples of GWAS in pathogens of crops (Plissonneau et al., 2017; Talas & McDonald, 2015). One of the challenges in using GWAS in fungi and oomycetes is the presence of population stratification and the long linkage disequilibrium observed in many pathogen species, which puts limits on the fine mapping of traits and markers (Bartoli & Roux, 2017). Application of GWAS in invasive insects is still in its infancy, but we expect that this approach will see rapid growth in the near future.

Landscape genomics aims to take advantage of the variation in natural populations to identify the environmental factors that shape adaptive variation and drive local adaptation. It is a promising approach to help predict adaptive variants in forest invasive species (Rellstab, Gugerli, Eckert, Hancock, & Holderegger, 2015). Genotype–environment association (GEA) methods aim to identify adaptive loci involved in local adaptation using correlations between genetic and environmental data (Forester, Lasky, Wagner, & Urban, 2018). Multivariate methods such as redundancy analysis provide a combination of low false‐positive and high true‐positive rates across all levels of selection (Forester et al., 2018). There are currently few examples of using these methods to detect genetic variation associated with adaptation in forest invasive species. In one such study, niche adaptation was hypothesized in fungal symbionts associated with the mountain pine beetle. The fungal species possessed distinct GEA signatures, with genetic variants associated with different environmental factors, including temperature and drought (Ojeda Alayon et al., 2017).

Association and landscape genomic studies can reveal candidate loci associated with traits. Understanding the mechanisms underlying the genotype–phenotype connection is a far more complex endeavor and requires an integrated approach that draws knowledge from diverse fields such as physiology, functional ecology, and genomics (de Villemereuil, Gaggiotti, Mouterde, & Till‐Bottraud, 2016; Morgan, Herman, Johnson, Olson, & Ungerer, 2018; Roe et al., 2018). Detailed exploration of the complex phenotypic variation within a range of environmental conditions (a.k.a. phenomics) must be conducted in parallel with genomic approaches (Bazakos, Hanemian, Trontin, Jimenez‐Gomez, & Loudet, 2017) to ensure adaptive phenotypes are appropriately identified. Furthermore, the genomic architecture of invasive phenotypes is often complex and likely polygenic in nature (Santure & Garant, 2018). Multifaceted approaches are needed to verify the causative function of identified loci to identify the relative contributions of gene regions to the observed phenotype (Cullingham et al., 2019), which can be challenging in nonmodel organisms. The greatest advances in adaptive genomics of invasion have been made with invasive plants (Colautti & Barrett, 2013; Richards, Schrey, & Pigliucci, 2012; Sultan, Horgan‐Kobelski, Nichols, Riggs, & Waples, 2013; Vandepitte et al., 2014), but we expect studies in this field on insects and pathogens in the near future.

There may be some low‐hanging fruits that will come to light with increased genome comparisons. For example, in several lineages of filamentous plant pathogens repeat‐driven genome expansions, in particular of genes encoding proteins involved in host interactions, generate genome plasticity that contributes to the emergence of new virulence traits (Raffaele & Kamoun, 2012; Sipos et al., 2017). An intriguing pattern is that codon optimization was related to the capacity of pathogens to colonize multiple hosts (Badet et al., 2017), a trait that could be important for invasiveness in fungi (Philibert et al., 2011). It is possible that simple genomic signatures can help predict traits related to invasiveness and potentially inform risk assessment. With continued growth in genomic technologies, we are now, more than ever, able to dissect the genomic contributions to phenotypic variation for invasive insects and pathogens and gain insight into the process of adaptive evolutionary change within invasive populations of forest pests.

## 4 FROM DNA TO DECISIONS

Genomic data collected during biosurveillance activities will only be useful to decision‐makers if a system is in place that allows the analysis outcomes to be coherently communicated and interpreted. In some cases, direct actions will ensue from the genomic analyses. The accurate identification of species that are regulated, for example, *Lymantria dispar* var *asiatica* or *P. ramorum* in North America or parts of Europe, would trigger an immediate response from regulatory authorities that could range from eradication to containment. When unknowns are intercepted, taxonomic placement provided by genomic data can be used to determine the threat level, strictly from a taxonomic perspective. For example, the discovery of a new unknown *Phytophthora* in a lineage that comprises aggressive pathogens would immediately raise some concern.

Estimating a probability of assignment to a source and pathway of invasion will likely not generate immediate actions but could guide trade negotiations as well as inform phytosanitary guidelines. Such knowledge will contribute to pathway risk assessment, one of the key steps to prevent future biological invasions (Hulme, 2009). Regulatory agencies have limited resources for inspections and surveys, and genomic‐based information can help prioritize these activities and concentrate efforts to goods arriving from high‐risk regions known to transmit potential invasives (Hulme, 2009). Additional knowledge about the geographic source combined with traits that are epidemiologically relevant (e.g., flight capacity, thermal limits, and host range) can contribute to assessing the risk of establishment and guide containment or eradication efforts.

### 4.1 Pipeline to success

**Figure 2:**
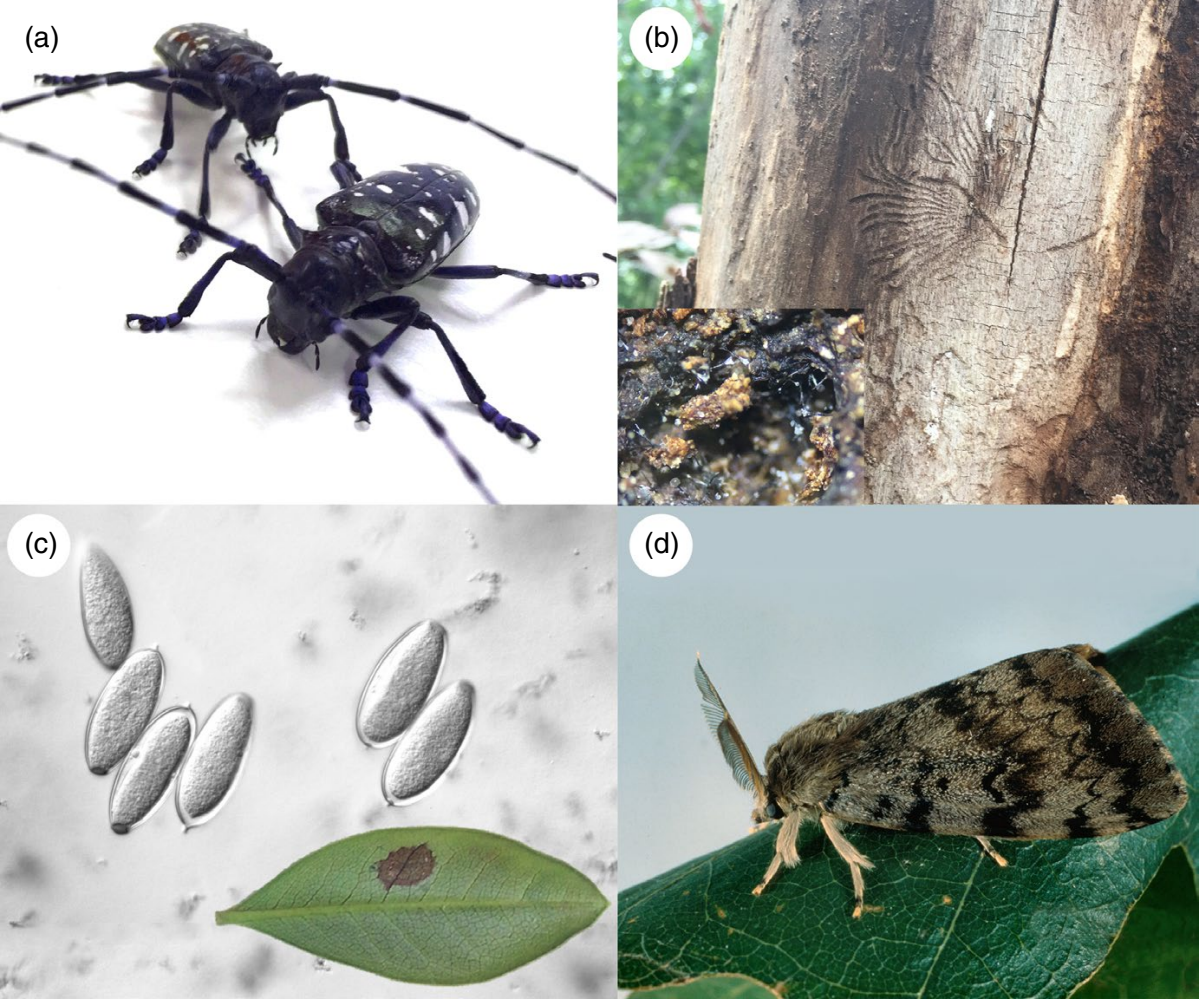
Four high‐risk forest invasives targeted for genomic biosurveillance. (a) Asian long‐horned beetle (*Anoplophora glabripennis*); (b) galleries of the American elm bark beetle (*Hylurgopinus rufipes*), the insect vector to the fungal agent responsible for Dutch elm disease (inset, synnemata of *Ophiostoma novo‐ulmi*); (c) *Phytophthora ramorum* sporangia (inset) and symptomatic European larch; and (d) Asian gypsy moth (*Lymantria dispar asiatica*)

Prior to invasion, often little is known of new invasive species. *Phytophthora ramorum* was not even known until landscape‐level tree mortality and shoot and leaf blight in nurseries were observed (Rizzo & Garbelotto, 2003; Werres et al., 2001). As such, genomic resources, identification tools, and basic biological information are often lacking, hampering timely management efforts. Developing a framework or pipeline to rapidly generate the knowledge and tools required would provide critical support to invasive species response efforts. Biosurveillance of Alien Forest Enemies (BioSAFE—http://www.biosafegenomics.com) is an initiative that is designed to rapidly generate genomic resources and tools for high priority forest invasive pests and pathogens (Figure 2) (Bilodeau et al., 2018; Roe et al., 2018). This interdisciplinary, multinational team of collaborators include entomologists, pathologists, bioinformaticians, modelers, economists, and regulators who aim to develop surveillance tools in diverse taxonomic groups (Coleoptera, Lepidoptera, Ascomycota, and Oomycota) of some of the most important pests and pathogens of trees: the Asian long‐horned beetle (*Anoplophora glabripennis*), Asian gypsy moth (*L. dispar*), Dutch elm disease (*O. novo‐ulmi*), and sudden oak death (*P. ramorum*).

**Figure 3:**
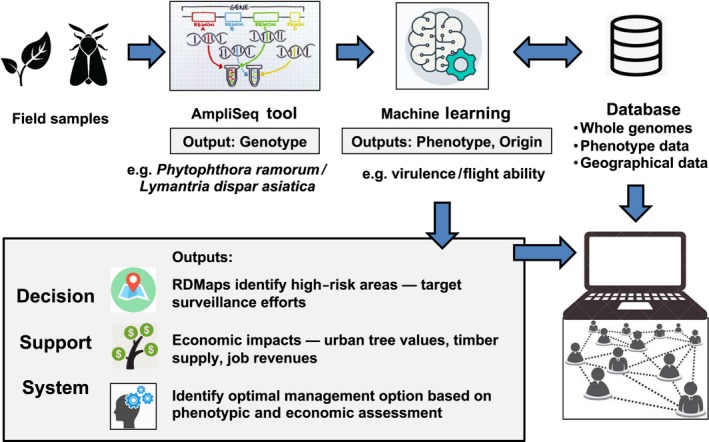
Proposed workflow of a pipeline for the BioSurveillance of Alien Forest Enemies (BioSAFE). Reproduced with modification courtesy of Pierre Bilodeau and Springer (Bilodeau et al., 2018)

This comprehensive biosurveillance pipeline aims to rapidly generate and operationalize genomic surveillance tools for accurate taxon identification, assignment of intercepted samples to putative sources, prediction of fitness traits that could impact invasion success, generation of risk and distribution maps, and a decision support system that can provide decision‐makers with a user‐friendly tool to interact with genomic data and predicted outcomes (Figure 3). This pipeline takes full advantage of the remarkable increase in genome sequencing capacity, combined with extensive phenotyping and bioinformatic analyses to rapidly fill knowledge gaps. Genomic biosurveillance of invasive species should generate transformative changes by speeding up and improving decision‐making and by informing mitigation and management in real‐time during outbreaks. Real‐time genomic epidemiology is already changing the way human infectious disease outbreaks are detected, characterized, and managed (Halachev et al., 2014; Tang & Gardy, 2014). Genome sequencing is becoming part of routine clinical diagnoses of human pathogens and helps predict infectious disease outcome and model transmission risks to inform authorities about disease prevention (Struelens & Brisse, 2013). We envision a similar framework designed to produce real‐time genomic biosurveillance of forest invasive enemies.

The BioSAFE pipeline consists of (a) genomic resource development; (b) diagnostic tool design; (c) predictive assignment of unknown samples; and (d) a decision support framework (Figure 3). The initial step is to develop the genomic resources to answer the key biological questions outlined above about identification and source. This requires assembling global collections of samples to generate global genomic profiles of the targeted species. These profiles can then be used to design tools, either with whole genomes or with a genome reduction approach, to identify taxa, geographic origin, and fitness traits using genome‐wide SNPs. Already genome‐derived tools have been designed for the identification of tree rust fungi and of Phytophthora species and lineages, and this is being applied in eradication programs targeting the EU1 lineage of *P. ramorum* in Oregon (Bergeron et al., 2019; Feau et al., 2016, 2018; Leboldus et al., 2019). Novel efficient and rapid algorithms using machine‐learning can provide a probability of assignment given the genomic reference library and associated metadata developed for each invasive species (Georges‐Filteau, Hamelin, & Blanchette, 2019). The outcome of the genomic predictions can then be integrated into a decision support system to provide a user with easily interpretable outcomes. To be useful, this genome‐wide population sequencing data and associated metadata must be in open‐source databases and publicly accessible, creating a foundation for real‐time genomic biosurveillance of forest invasive enemies.

## 5 FUTURE OUTLOOKS

### 5.1 From benchtop to tailgate—Molecular identification in the wild

Most current molecular techniques are still shackled to the laboratory benchtop. Our current vision of genomic biosurveillance is aimed to be conducted, at least in part, in a laboratory environment. Increasingly, there is a need for mobile diagnostic or “point‐of‐need” (PON) approaches. This is particularly relevant for biosurveillance of forest invasive species because sampling is often conducted in remote locations, increasing both the time from collection to answer and the speed of action when a positive is found. Portable, real‐time molecular‐based field identification of forest pests is a critical, yet unrealized, step in genomic biosurveillance.

**Figure 4:**
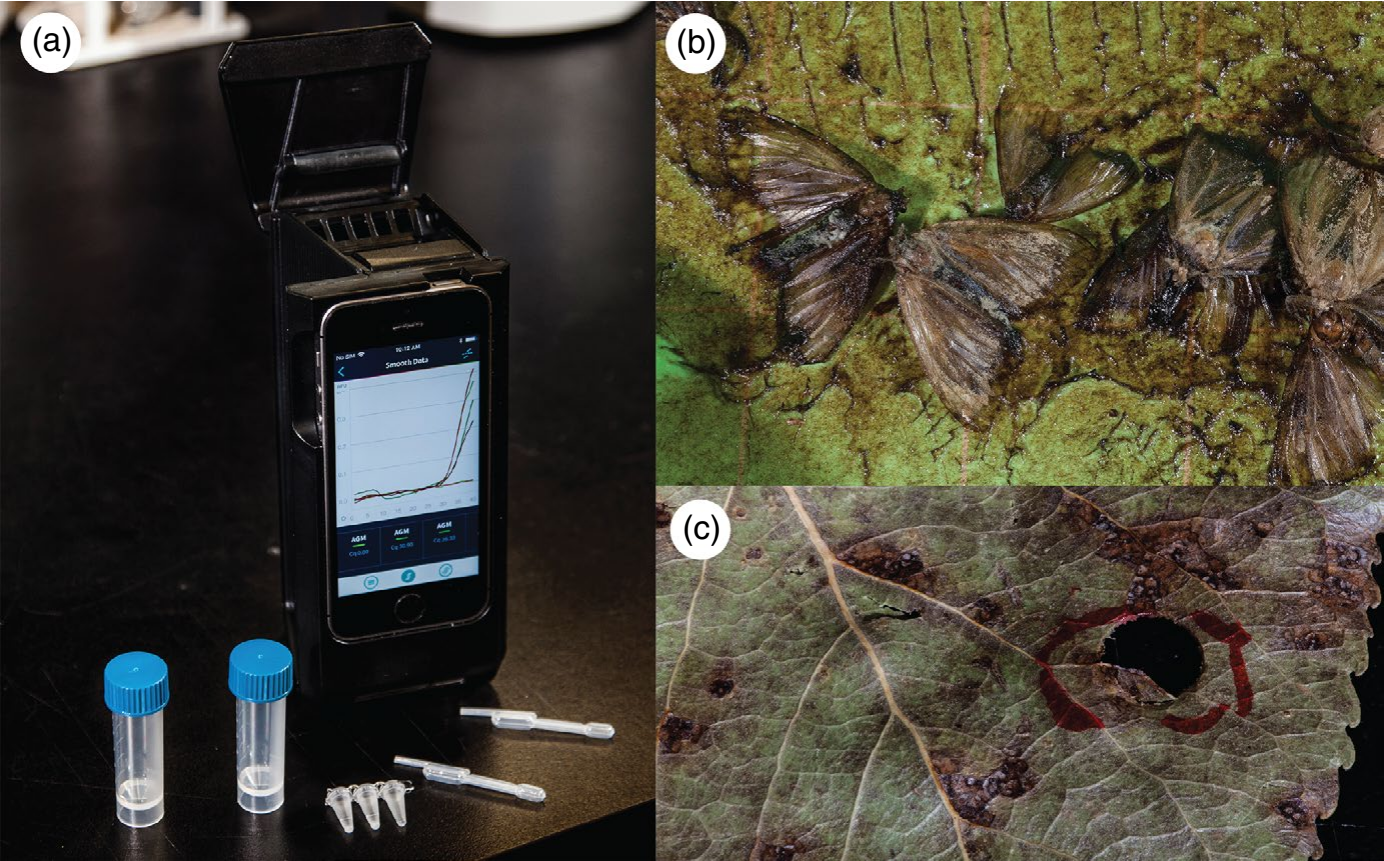
Field portable DNA‐based detection of insect and pathogen invasive species. (a) Handheld Biomeme™ real‐time PCR device, shelf‐stable reagents, and the disposable pipettes required to perform field qPCR; (b) Gypsy moth (*Lymantria dispar*) samples captured in pheromone traps and used for PCR identification (Stewart et al., 2016); and (c) Poplar leaf tested for the presence of the Septoria canker pathogen, *Sphaerulina musiva* (Herath et al., 2016)

The field of PON diagnostics is rapidly changing as new techniques and technologies are developed, mostly driven by medical applications (Yager, Domingo, & Gerdes, 2008). A number of PON approaches are actively being developed to support field‐based tests that provide rapid sample‐to‐answer results. In general, diagnostic assays have three basic steps: nucleic acid extraction, target DNA amplification, and genetic variant detection. To achieve a fully mobile diagnostic unit, these steps must be simplified, miniaturized, integrated, and/or eliminated from the workflow. With increasing demands for portable molecular assays, commercial nucleic acid detection kits have become available that use membranes or magnetic beads to capture and separate nucleic acids from impurities. For example, cellulose‐based techniques are showing promise in simplifying the extraction process and incorporating it into a field‐based or PON workflow that is rapid and inexpensive (Gerbers, Foellscher, Chen, Anagnostopoulos, & Faghri, 2014; Zou et al., 2017), ideal for portable diagnostics. DNA amplification, the next step within a diagnostic workflow, still relies mostly on PCR. Although most PCRs are still conducted in bulky tabletop instruments, new portable devices have been developed such as miniPCR™ or microfluidic “lab on a chip” devices that have liberated PCR techniques from the laboratory. Technologies that achieve complete portability are now being developed. Handheld quantitative PCR (qPCR) devices (e.g., Biomeme™) have modified existing qPCR assays to work with shelf‐stable reagents so that the entire process can be in the field (Figure 4). Alternative PCR‐free assays avoid pitfalls of inhibition from environmental contaminants and are gaining popularity. Loop‐mediated isothermal amplification is one assay method that is currently being used in PON scenarios, including detection of invasives at ports of entry (Blaser et al., 2018; Notomi et al., 2000). We expect that new technologies will rapidly evolve to fill the need for PON diagnostics, providing much needed field support for the management of invasive species.

Field‐based genome sequencing or target enrichment approaches could be even more powerful, by providing an unbiased method that requires no *a priori* knowledge of the pest or pathogen, thus combining diagnostics, identification, and genome characterization in a single operation. It is already revolutionizing the field of plant virology where it is becoming a frontline diagnostic tool (Adams, Fox, Boonham, Massart, & Jonghe, 2018). High‐throughput sequencing of infected plants has been applied to discover the entire viral genome of an unknown pathogen (Adams et al., 2009). Viruses are a low‐hanging fruit because of their small genome, but with the increasing sequencing capacity, this approach can be envisaged for other types of pathogen and pests. Already, field pathogenomics, sequencing RNA of infected crop leaves to obtain and extract sequence data, shows great promises to predict shifts in pathogen populations and the development of new pathogenic races in agricultural crops (Hubbard et al., 2015).

Ultimately, combining unbiased shotgun or targeted sequencing with portability is the key to future genomic biosurveillance. The MinION (Oxford Nanopore Technologies) is a sequencing technology that shows promises for field applications. It relies on single‐molecule sequencing in a compact flow cell that can be powered from a laptop computer. The MinION can generate sequence data in real‐time under field conditions. Although it is not yet fully portable, it has been used to monitor disease outbreaks in field‐based clinics or rapidly deployable laboratories (Quick et al., 2016, 2017; Walter et al., 2017), to perform real‐time field sequencing of crop viruses (Boykin et al., 2018; Shaffer, 2019), to conduct biodiversity surveys in field settings (Menegon et al., 2017; Pomerantz et al., 2018) and even to sequence genomes on the International Space Station (Castro‐Wallace et al., 2017). Such field sequencing methods are in their infancy and still suffer from major challenges with regard to nucleic acid isolation and library construction. This will be even more complicated in genomic biosurveillance of forest invasive species, given the complex nature of the samples to be processed (different hosts, life stages, tissues). Another major hurdle will be the bioinformatic analyses that have to be performed for comparison to existing external databases and internet connectivity, currently required for analyses (Quick et al., 2016). Yet, portable field genome sequencing is a key example of how genomic approaches can be implemented outside of a traditional laboratory environment to provide real‐time genomic data for biosurveillance.

### 5.2 Reading from a different code

Over the past 10 years, there is increasing evidence that points to the importance of alternative modes of inheritance in determining an organism's phenotype—a story written in a different code. Transgenerational inheritance, or epigenetics, is a nongenetic form of inheritance that can influence phenotypes in response to environmental conditions (Bonasio, 2015; Verhoeven, vonHoldt, & Sork, 2016). There are a number of mechanisms to explain this phenomenon (Tikhodeyev, 2018), but DNA methylation has received the most focus (Glastad, Hunt, & Goodisman, 2019; Verhoeven et al., 2016). In invasive species, epigenetic mechanisms have been proposed as a nongenetic means to facilitate adaptation to new environments (Hawes et al., 2018; Huang et al., 2017). While much of the work on epigenetics and DNA methylation is still in its infancy, some early results from invasive species hint at larger adaptive processes that promote rapid phenotypic evolution over short time scales (Danchin, Pocheville, Rey, Pujol, & Blanchet, 2019; Ni et al., 2018; Schrey et al., 2016; Stapley, Santure, & Dennis, 2015). For example, invasive species often experience genetic bottlenecks and a loss of genetic diversity over the course of an invasion, but without the expected loss of fitness. Epigenetic inheritance has been proposed as a means to compensate for low genetic diversity by providing heritable phenotypes that can survive in new habitats (e.g., Richards et al., 2012). Linking signatures of epigenetic inheritance to fitness in invasive species is promising and could be critical for prevention and management (Hawes et al., 2018). Emerging examples of epigenetic markers being developed into diagnostic monitoring tools (Ardura, Clusa, Zaiko, Garcia‐Vazquez, & Miralles, 2018; Eirin‐Lopez & Putnam, 2019). We expect that, with further study, these novel inheritance mechanisms will generate a wealth of knowledge about invasions and could provide fertile ground for a new generation of biosurveillance tools.

## 6 CONCLUSION

A major shift occurred when genomics transitioned from a specialized science restricted to a handful of model organisms to a diverse field exploring the infinite complexity of the biological world. This shift from model to nonmodel organisms is having a substantial impact in the field of forest invasive species. Genomics is becoming part of the invasive species management toolbox by providing accurate diagnostics, identification of sources and pathways, and foundational knowledge on which to base risk assessments. Technological advancements have brought genomic data within reach of large communities of researchers for these nonmodel organisms. The next challenge will be to make these tools available and relevant to the community that needs it, that is, the end users and managers of resources threatened by forest invasive species. Despite the potential benefits genomic tools bring to invasive species management, there has been limited adoption within the end‐user community. Bilodeau et al. (2018) provide an in‐depth examination of the potential barriers to adoption within an operational environment. These include end‐user knowledge of genomics, the effectiveness and efficiency of genomic tool development, and cost effectiveness. Including end users in the development of genomic biosurveillance tools ensures usability and adoption at the end of the development pipeline. The multifaceted nature of the BioSAFE pipeline (Bilodeau et al., 2018; Roe et al., 2018) is a critical aspect of our approach and requires effective collaborative networks to assist with sample collection, data production, analysis, and tool implementation. Involving end users will ensure that we develop tools that provide the knowledge necessary to direct management or regulatory decisions and to increase the probability of technological adoption (Bilodeau et al., 2018). Pairing the results of genomic tools with a decision support system will help guide complex decisions involving invasive species management and control.

Cost can be a critical barrier to the development and implementation of genomic technology (Crann, Fairley, Badulescu, Mohn, & O’Doherty, 2015). While sequencing costs are continually decreasing, the costs of other aspects of the genomic pipeline have maintained or increased (Sboner, Mu, Greenbaum, Auerbach, & Gerstein, 2011). Although a valuable exercise (Porth et al., 2015), a detailed economic analysis is beyond the scope of this paper. It is important, however, to place these costs into context. It is estimated that the emerald ash borer, a single invasive forest insect, has cost the North American economy billions of dollars (McKenney et al., 2012; The cost of research and development of genomic biosurveillance tools for four high‐risk invasives (Figure 2) targeted by the BioSAFE initiative represents a small fraction of the overall costs that these or other invasive forest pests pose to society. With the rapidly changing landscape of genomic technologies, we expect that solutions to many of the existing challenges will be realized. We eagerly anticipate the continued evolution of the rapidly changing field of invasive species genomics and the support it provides to the management of invasive species.

## CONFLICT OF INTEREST

None declared.

## Data Availability

Data sharing is not applicable to this article as no new data were created or analyzed in this study.
